# Translation and concurrent validity, sensitivity and specificity of Chinese version of Short Orientation Memory Concentration Test in people with a first cerebral infarction

**DOI:** 10.3389/fnhum.2023.977078

**Published:** 2023-06-01

**Authors:** Jiang-Li Zhao, Pei-Ming Chen, Shamay S. M. Ng, Yu-Rong Mao, Dong-Feng Huang

**Affiliations:** ^1^Department of Rehabilitation Medicine, The First Affiliated Hospital, Sun Yat-sen University, Guangzhou, Guangdong, China; ^2^Department of Rehabilitation Sciences, The Hong Kong Polytechnic University, Hong Kong, Hong Kong SAR, China; ^3^Department of Rehabilitation Medicine, The Seventh Affiliated Hospital, Sun Yat-sen University, Shenzhen, Guangdong, China

**Keywords:** rehabilitation, stroke, cognition, Short Orientation Memory Concentration Test (SOMC), validity, sensitivity, specificity

## Abstract

**Purpose:**

This study aimed to translate the English version of the Short Orientation-Memory-Concentration (SOMC) test into a Chinese version, denoted the C-SOMC test, and to investigate the concurrent validity, sensitivity, and specificity of the C-SOMC test against a longer and widely used screening instrument in people with a first cerebral infarction.

**Methods:**

An expert group translated the SOMC test into Chinese using a forward–backward procedure. Eighty-six participants (67 men and 19 women, mean age = 59.31 ± 11.57 years) with a first cerebral infarction were enrolled in this study. The validity of the C-SOMC test was determined using the Chinese version of Mini Mental State Examination (C-MMSE) as the comparator. Concurrent validity was determined using Spearman’s rank correlation coefficients. Univariate linear regression was used to analyze items’ abilities to predict the total score on the C-SOMC test and the C-MMSE score. The area under the receiver operating characteristic curve (AUC) was used to demonstrate the sensitivity and specificity of the C-SOMC test at various cut-off values distinguishing cognitive impairment from normal cognition.

**Results:**

The total score for the C-SOMC test and the score for item 1 on this test exhibited moderate-to-good correlations with the C-MMSE score, with respective ρ-values of 0.636 and 0.565 (*P <* 0.001). The scores for each of items 2, 4, 5, 6, and 7 yielded fair correlations with C-MMSE score, with ρ-value from 0.272 to 0.495 (*P <* 0.05). The total score on the C-SOMC test and the item score were good predictors (adjusted *R*^2^ = 0.049 to 0.615) of the C-MMSE score, and six items were good predictors (adjusted *R*^2^ = 0.134 to 0.795) of the total score. The AUC was 0.92 for the C-SOMC test. A cut-off of 17/18 on the C-SOMC test gave optimal performance: correct classification of 75% of participants, with 75% sensitivity and 87.9% specificity.

**Conclusion:**

The C-SOMC test demonstrated good concurrent validity, sensitivity and specificity in a sample of people with a first cerebral infarction, demonstrating that it could be used to screen for cognitive impairment in stroke patients.

## Introduction

Stroke is one of the leading causes of death and disability worldwide (Stinear et al., 2020), and the number of stroke survivors is increasing as populations increase and age (Li, 2020). Cognitive impairment is a common comorbidity following stroke (Swartz et al., 2016), affecting approximately 20% to 80% survivors (Sun et al., 2014; Zhu et al., 2022). Moreover, up to 78% of acute stroke patients show impairments in at least one cognitive domain after stroke (Lesniak et al., 2008), and subacute and chronic stroke patients commonly show deficits in attention, executive functioning, mental processing speed, visual perception, construction ability, memory, and language expression (Stephens et al., 2004). Post-stroke cognitive impairment directly affects patients’ ability to understand, learn, and implement their rehabilitation treatment plan, resulting in poor recovery of other stroke-affected functions, such as motor function, swallowing function, and speech function (Yuan et al., 2021). Furthermore, post-stroke cognitive impairment often restricts patients’ abilities to perform daily activities and participate in social activities, leading to a reduced quality of life (Ye et al., 2022). Cognitive impairment is also associated with recurrence of ischemic stroke in high-risk patients despite adequate medical therapy, including antiplatelet therapy (Kwon et al., 2020). Consequently, post-stroke cognitive impairment also imposes a heavy burden on families and society (Crichton et al., 2016), underscoring the need for timely detection and symptomatic treatment to aid rehabilitation. Validated screening instruments, such as the Short Orientation-Memory-and Concentration (SOMC) test, can be used for early detection of cognitive impairment, as such brief and easily administered screens are suitable for use with stroke patients (Borson et al., 2003).

Although the Mini Mental State Examination (MMSE) is the most widely known and utilized cognitive impairment instrument, it is time consuming and its acceptability by non-psychiatrists has been questioned (Borson et al., 2003). Furthermore, it contains a drawing task, which is difficult for stroke patients with upper limb paralysis to complete. In contrast, the SOMC test is a brief, verbally administered screen that does not require the patient to write or draw (Goring et al., 2004). As mentioned, this makes the SOMC test suitable for use with stroke patients, and it is also one of the most commonly administered screens for assessing cognitive function in a variety of populations (Fillenbaum et al., 1998), such as healthy older adults (Hanlon et al., 1998; Stutts et al., 1998; Cohen-Mansfield and Perach, 2012), those with dementia (Boucher et al., 1996), Alzheimer’s disease (Mavioglu et al., 2006), hip fracture (Bech et al., 2015), multiple sclerosis (Vlaar and Wade, 2003), people exposed to anesthesia (Bilotta et al., 2009; Bronco et al., 2010; Werner et al., 2015), and older adults with cancer (Jayani et al., 2020). To the best of our knowledge, few studies have assessed the use of the SOMC test in stroke patients. Wade and Vergis (1999) reported that the SOMC test was reasonably effective for screening for major cognitive deficits. Katzman et al. (1983) proved that the SOMC test could discriminate between mild, moderate, and severe cognitive deficits, and Queally et al. (2010) reported that the moderate-impairment cut-off of the SOMC test was scored more accurately than that of the MMSE. Therefore, the SOMC test may have great potential for detecting cognitive impairment in people with stroke.

Brief screening instruments such as the SOMC test are lacking in China, and there has been no report of a translation of the SOMC test into Chinese. Accordingly, the aim of this study was to translate the SOMC test into Chinese and explore its concurrent validity, sensitivity, and specificity in people with stroke.

## Materials and methods

### Translation process

We employed a forward-backward procedure to translate the SOMC test from English into Chinese (Beaton et al., 2000) and denoted this translation the C-SOMC test. An expert committee comprising the principal investigator, four translators (two Chinese native speaker and two English native speakers), two experienced physiotherapists, two occupational therapists and two rehabilitation physicians reviewed all of the versions of the C-SOMC test to ensure its smooth completion. The committee developed the final version of the C-SOMC test for field testing (Beaton et al., 2000). We revised item 3/7 and 6 to adapt them to suit Chinese culture. Specifically, we replaced names and addresses in item 3/7 with those that are common in Chinese culture, as follows: Arthur/Jones, Joe/Smith, Tom/White, and Philip/Winter were replaced with Li Wei, Wang Jun, Zhang Hua, and Liu Bo, respectively; West Street, Church Road, Station Road, and North Way were replaced with Tian He Road, Hong Qiao Road, He Ping Road, Chang An Street, respectively; and Witney, Banbury, Aylesbury, Oxford were replaced with Guangzhou, Shanghai, Tianjin, and Beijing, respectively. Due to cultural differences, it was challenging to find a task equivalent to that of item 6, which involves speeking the 12 months of the year in reverse order. This is because in Chinese culture, this task involves counting backward from 12 to 1, which is similar to the part of item 5 (which involves counting backward from 20 to 1). Ultimately, as this task needs to be based on stating 12 well-known words in a certain order, we used the twelve Chinese zodiac signs (TCZS) which are familiar to everyone in China as a substitute for the 12 months of the year. The TCZS are, in order, rat, cow, tiger, rabbit, dragon, snake, horse, sheep, monkey, chicken, dog, and pig. Although some people may only know some of the order and cannot quickly state all TCZS, every Chinese person knows all of them. Thus, requiring the TCZS to be spoken in reverse order was the most appropriate substitute for requiring the 12 months of the year to be spoken in reverse order. The C-SOMC test is presented in Supplementary Appendixs 1, 2.

### Scoring

The total score of C-SOMC test ranges from 0 to 28, as does that for the SOMC test, which comprises six items and was first reported by Katzman et al. (1983). We scored the C-SOMC test such that compared with lower scores, higher scores indicate better cognitive function (Tieges et al., 2015). For item 1 (“What year is it now?”), 4 points are awarded for a correct answer, while 0 points are awarded for an incorrect answer. For item 2 (“What month is it now?”), 3 points are awarded for a correct answer, while 0 points are awarded for an incorrect answer. For item 4 (“About what time is it? within an hour”), 3 points are awarded for a correct answer, while 0 points are awarded for an incorrect answer. For item 5 (“Count backward 20 down to 1”), 4 points are awarded for completion with no errors, 2 points are awarded completion with one error, and 0 points are awarded for completion with two or more errors. For item 6 (“State the TCZS in reverse order”), 4 points are awarded for completion with no errors, 2 points are awarded completion with one error, and 0 points are awarded for completion with two or more errors. For item 3/7 (“Repeat the address given”), 2 points are subtracted from a score of 10 for each error on a first name, family name, number, road name or city name, so the score ranges from 0 to 10 points. Furthermore, because some people may be unable to state all TCZS in reverse order, we also removed item 6 and treated the remaining five items (i.e., item 1, 2, 4, 5, and 3/7) as a shorter C-SOMC test, denoted the C-SOMC-R6 test.

### Subjects

In a previous study with a similar design, a sample size of 38 was sufficient to determine the concurrent validity of the SOMC test (Wade and Vergis, 1999). To draw more robust conclusions, this study enrolled 86 in-patients with a first cerebral infarction in the Department of Rehabilitation Medicine of the First Affiliated Hospital of Sun Yat-sen University in China from August 2015 to July 2019. The inclusion criteria were (Stinear et al., 2020) the occurrence of a first stroke with unilateral hemiparetic lesions confirmed by magnetic resonance imaging or computed tomography; (Li, 2020) an interval of ≥5 days after stroke; (Swartz et al., 2016) age of 18–80 years; (Zhu et al., 2022) a Glasgow Coma Scale ≥ 12; (Sun et al., 2014) no severe deficits in communication; and Lesniak et al. (2008) ability to give informed consent. The exclusion criteria were (1) an inability to complete assessments due to medical instability; (2) diagnosis with other neurological diseases that may affect cognitive function; or (3) a history of medication for mental illness.

The participants’ demographic details and major comorbidity data were collected from medical records. Their demographic information is shown in Table 1. This study was approved by the Human Subjects Ethics Subcommittee of the First Affiliated Hospital of Sun Yat-sen University in China, and informed written consent was obtained from all of the participants.

**TABLE 1:** Characteristics of the study participants.

Variable	Values		
	**All (*n* = 86)**	**Group 1 (*n* = 66)**	**Group 2 (*n* = 20)**
**Sex**			
Male	67 (77.91)	54 (81.82)	13 (65.00)
Female	19 (22.09)	12 (18.18)	7 (35.00)
**Stroke type**			
Ischemic	86 (100.00)	66 (100.00)	20 (100.00)
**Affected side**			
Right	48 (55.81)	30 (45.45)	8 (40.00)
Left	38 (44.19)	36 (54.55)	12 (60.00)
**Cognition**			
Normal)	66 (76.74)	66 (100.00)	
Mild	14 (16.28)		14 (70.00)
Severe	6 (6.98)		6 (30.00)
Age (years)	59.31 ± 11.57 (40-79)	57.36 ± 11.55 (40-79)	65.75 ± 9.24 (47–79)
Onset (days)	29.40 ± 16.87 (5–87)	27.36 ± 16.45 (5–87)	36.10 ± 16.88 (15-62)
Education (years)	9.35 ± 4.38 (0–21)	10.09 ± 4.14 (5–21)	6.90 ± 4.36 (0–15)

Values are *n* (%) or Mean ± SD (range). Normal: Chinese version of the Mini Mental State Examination, 24–30. Mild: Chinese version of the Mini Mental State Examination, 18–23. Severe: Chinese version of the Mini Mental State Examination, 0–17.

### Procedure

Prior to collecting baseline data, an experienced physiotherapist with more than 10 years of clinical experience in stroke rehabilitation was trained to correctly administer the C-SOMC test and the C-MMSE properly. The two measures were conducted in a certain order which was first the C-MMSE and then the C-SOMC test. A sufficient rest period was provided during the measurement procedure to prevent fatigue affecting the results. The entire procedure took approximately10–15 min.

### Outcome measures

#### SOMC test

The SOMC test is a 6-item orientation–memory-concentration test with a total score that ranges from 0 to 28 (Katzman et al., 1983). The score is highly correlated (*r* = 0.92) with and nearly as sensitive as the full orientation-memory-concentration test (Nerz et al., 2017). Furthermore, the score on the SOMC test is correlated with the immediate (Pearson’s *r* = 0.68) and delayed (Pearson’s *r* = 0.74) recall scores for the paragraph from the Rivermead Behavioral Memory Test (Wade and Vergis, 1999). Compared with lower scores, higher scores on the SOMC test indicate better cognitive function (Tieges et al., 2015). The suggested scores for categorization are as follows: 24–28 = normal cognition; 19–23 = possible impairment; ≤18 = dementia (Morris et al., 1989).

#### MMSE

The MMSE is the most widely known and utilized cognitive impairment instrument. It consists of 11 questions, takes approximately 10 min to administer, and has a total score ranging from 0 to 30 (Lampley-Dallas, 2001). A normal MMSE score varies depending on whether any adjustments are made for education alone or both education and age (Crum et al., 1993). For instance, without any adjustments, a score of 24 to 30 is considered to be normal, a score of 18 to 23 is considered consistent with mild dementia, and a score of 0 to 17 is considered consistent with severe dementia (Tombaugh and McIntyre, 1992).

### Statistical analysis

#### Participants

The demographic and clinical characteristics of the participants in this study (*n* = 86) were interpreted using descriptive statistics.

### Validity

The concurrent validity of the C-SOMC test was assessed by calculating the correlations between the C-SOMC test scores and the C-MMSE scores. The Spearman’s rank correlation coefficient (ρ) was used to evaluate these correlations. ρ-value from 0 to 0.25, 0.25 to 0.50, 0.50 to 0.75, and >0.75 were considered to indicate weak, fair, moderate-to-good, and good-to-excellent concurrent validity, respectively (Portney, 2009). If there was a significant correlation between items and the total score on the C-SOMC test, linear regression was performed with the “enter” method to examine what proportion of C-SOMC scores could be explained by each item (Seber and Lee, 2012).

### True positive and true negative

The number of true positive and true negative cases were calculated using three cut-offs (at 18/19, 17/18, and 16/17) on the C-SOMC test and two cut-offs (at 23/24 and 24/25) on the C-MMSE. The participants who scored below both cut-offs on the C-SOMC test and the C-MMSE were considered true positives, whereas the participants who scored above both cut-offs on the C-SOMC test and the C-MMSE were considered true negatives.

### Sensitivity and specificity

The MMSE was used as a comparator to measure the sensitivity and specificity of the C-SOMC test. The area under the receiver operating characteristic (ROC) curve [(area under the curve (AUC)] was calculated for the C-SOMC test. An ROC curve was used to demonstrate the sensitivity and specificity of the C-SOMC test for every possible cut-off value to distinguish cognitive impairment from normal cognition.

All of the statistical analyses were performed using SPSS version 20.0, with all of the tests being two-tailed. The level of significance was set as *P <* 0.05.

## Results

### Demographics

Eighty-six participants (67 men and 19 women) with a first cerebral infarction were enrolled in this study. The demographic and clinical characteristics of the participants are provided in Table 1.

### Outcome scores

The data in this study were not normally distributed, as determined by the Shapiro–Wilk test. The C-SOMC test and C-MMSE outcome scores are shown in Table 2.

**TABLE 2:** The participants’ scores for the outcome measures.

Variable	Values		
	**All (*n* = 86)**	**Group 1 (*n* = 66)**	**Group 2 (*n* = 20)**
C-SOMC	19.78 ± 6.10 (0–28)	22.00 ± 3.88 (10–28)	12.45 ± 6.45 (0–22)
C-SOMC-1	3.35 ± 1.49 (0–4)	3.82 ± 0.84 (0–4)	1.80 ± 2.04 (0–4)
C-SOMC-2	2.51 ± 1.11 (0–3)	2.86 ± 0.63 (0–3)	1.35 ± 1.53 (0–3)
C-SOMC-4	2.69 ± 0.92 (0–3)	2.95 ± 0.37 (0–3)	1.80 ± 1.51 (0–3)
C-SOMC-5	3.51 ± 1.23 (0–4)	3.70 ± 0.94 (0–4)	2.90 ± 1.77 (0–4)
C-SOMC-6	0.51 ± 1.31 (0–4)	0.67 ± 1.46 (0–4)	0.00 ± 0.00 (0–0)
C-SOMC-3/7	7.21 ± 3.15 (0–10)	8.00 ± 2.67 (0–10)	4.60 ± 3.25 (0–10)
C-SOMC-R6	19.27 ± 5.74 (0–24)	21.33 ± 3.50 (10–24)	12.45 ± 6.45 (0–22)
C-MMSE	25.59 ± 4.25 (11–30)	27.48 ± 1.93 (24–30)	19.35 ± 3.82 (11–23)

Values are Mean ± SD (range). C-SOMC, Chinese version of the Short Orientation-Memory-Concentration Test; C-SOMC-1, item 1 of the C-SOMC test; C-SOMC-2, item 2 of the C-SOMC test; C-SOMC-4, item 4 of the C-SOMC test; C-SOMC-5, item 5 of the C-SOMC test; C-SOMC-6, item 6 of the C-SOMC test; C-SOMC-3/7, item 3/7 of the C-SOMC test; C-SOMC-R6, five items (i.e., item 1, 2, 4, 5, and 3/7) of the C-SOMC test except item 6 constituted a shorter C-SOMC test; C-MMSE, Chinese version of the Mini Mental State Examination.

### Concurrent validity

**FIGURE 1:**
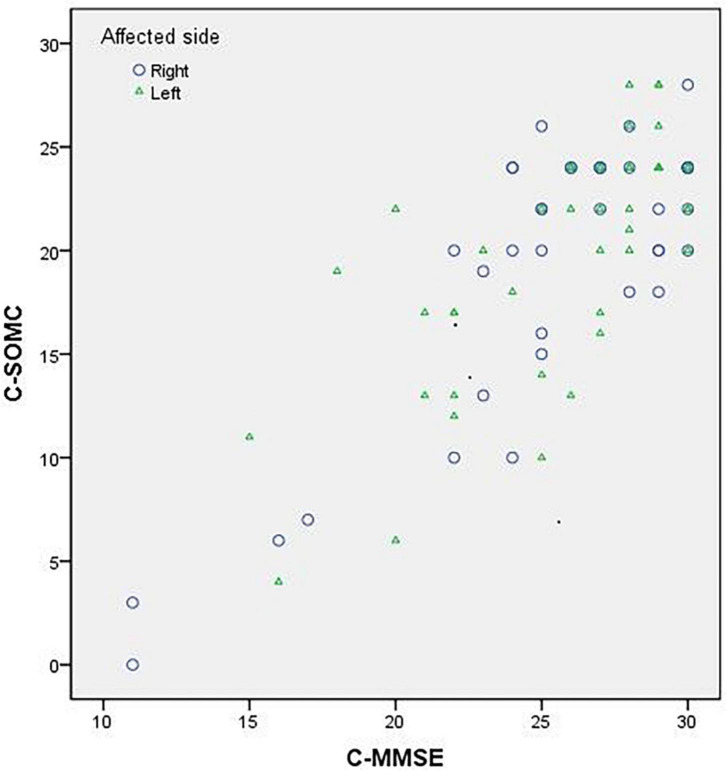
Relationship between the participants’ (*n* = 86) performance in the C-SOMC test and their performance in the C-MMSE.

The data of the participants were pooled to calculate the concurrent validity. The total score on the C-SOMC test and the C-MMSE score yielded a moderate-to-good correlation correlation (ρ = 0.636, *P <* 0.001). The score on item 1 of the C-SOMC test and the C-MMSE score also exhibited a moderate-to-good correlation (ρ = 0.565, *P <* 0.001). The scores for items 2, 4, 5, 6, and 3/7 of the C-SOMC test each generated a fair correlation with the C-MMSE score (ρ = 0.272– 0.495, *P <* 0.05), whereas the score for item 6 of the C-SOMC test yielded the lowest ρ-value. Furthermore, the total score for the C-SOMC-R6 test and the C-MMSE score exhibited a moderate-to-good correlation (ρ = 0.624, *P <* 0.001). The scores for five items on the C-SOMC test (items 1, 2, 5, 6, 3/7) each generated a moderate-to-good correlation with the total score on this test (ρ = 0.501 – 0.795, *P <* 0.001), whereas the score for item 4 exhibited a fair correlation with the total score on this test (ρ = 0.430, *P <* 0.001). The total score on the C-SOMC test and the scores for most of its items were good predictors (adjusted *R*^2^ = 0.214 to 0.615) of the C-MMSE, the exception was the score for item 6 (adjusted *R*^2^ = 0.049). The scores for each of six items on the C-SOMC test were good predictors of the total score on this test (adjusted *R*^2^ = 0.134 to 0.795), with the score for item 6 producing the lowest adjusted *R*^2^ value. Figure 1 shows the relationship between the participants’ performance on the C-SOMC test and their performance on the C-MMSE.

We next divided the participants into two groups: group 1 comprised 66 participants (54 men and 12 women) with normal cognition (an C-MMSE score ≥ 24), and group 2 comprised 20 participants (13 men and 7 women) with abnormal cognition (0 ≤ C-MMSE score ≤ 23). We separately analyzed the two groups for concurrent validity. The results showed that the group 1 did not exhibit correlations as good as those of the total pool of participants (*n* = 86), whereas group 2 exhibited similar correlations to those of the total pool of participants.

Detailed results of the validity analyses are summarized in Tables 3, 4.

**TABLE 3:** Correlations between the C-SOMC test scores and the C-MMSE scores in the stroke participants.

	All (*n* = 86)				Group 1 (*n* = 66)				Group 2 (*n* = 20)			
	**C-MMSE**		**C-SOMC**		**C-MMSE**		**C-SOMC**		**C-MMSE**		**C-SOMC**	
	**ρ**	***P***	**ρ**	***P***	**ρ**	***P***	**ρ**	***P***	**ρ**	***P***	**ρ**	***P***
C-SOMC	0.636^a^	0.000			0.313^b^	0.011			0.644^a^	0.002		
C-SOMC-1	0.565^a^	0.000	0.612 ^a^	0.000	0.300^b^	0.015	0.334^b^	0.006	0.567^a^	0.009	0.796^a^	0.000
C-SOMC-2	0.473^b^	0.000	0.538 ^a^	0.000	0.178^c^	0.153	0.336^b^	0.006	−0.151^c^	0.526	0.219^c^	0.354
C-SOMC-4	0.432^b^	0.000	0.430 ^b^	0.000	−0.020^c^	0.875	0.091^c^	0.465	0.540^a*^	0.014	0.498^b^	0.026
C-SOMC-5	0.324	0.002	0.516 ^a^	0.000	0.215^c^	0.083	0.455^b^	0.000	0.583^a^	0.007	0.734^a^	0.000
C-SOMC-6	0.272^b^	0.011	0.501 ^a^	0.000	0.167^c^	0.180	0.526^a^	0.000	E	E	E	E
C-SOMC-3/7	0.495^b^	0.000	0.795 ^a^	0.000	0.244^c^	0.048	0.731^a^	0.000	0.448^b^	0.048	0.728^a^	0.000
C-SOMC-R6	0.624^a^	0.000	0.933 ^a^	0.000	0.307^b^	0.012	0.856^a^	0.000	0.644^a^	0.002	1.000^a^	0.000

ρ-values indicate correlation coefficients by Spearman’s rank correlation coefficient.

^a^Good correlation.

^b^Fair correlation.

^c^Weak correlation. *P* < 0.05 indicates significant correlations. C-SOMC, Chinese version of the Short Orientation-Memory-Concentration Test; C-SOMC-1, item 1 of the C-SOMC test; C-SOMC-2, item 2 of the C-SOMC test; C-SOMC-4, item 4 of the C-SOMC test; C-SOMC-5, item 5 of the C-SOMC test; C-SOMC-6, item 6 of the C-SOMC test; C-SOMC-3/7, item 3/7 of the C-SOMC test; C-SOMC-R6, five items (i.e., item 1, 2, 4, 5, and 3/7) of the C-SOMC test except item 6 constituted a shorter C-SOMC test; C-MMSE, Chinese version of the Mini Mental State Examination. E:Because there is at least one constant, it cannot be statistics.

**TABLE 4:** Univariate linear regression of the C-SOMC test scores and the C-MMSE scores in the participants.

	All (*n* = 86)				Group 1 (*n* = 66)				Group 2 (*n* = 20)			
	**C-MMSE**		**C-SOMC**		**C-MMSE**		**C-SOMC**		**C-MMSE**		**C-SOMC**	
	**Adjusted R^2^**	**P**	**Adjusted R^2^**	**P**	**Adjusted R^2^**	**P**	**Adjusted R^2^**	**P**	**Adjusted R^2^**	**P**	**Adjusted R^2^**	**P**
C-SOMC	0.615	0.000			0.153	0.001			0.518	0.000		
C-SOMC-1	0.457	0.000	0.561	0.000	0.089	0.009	0.177	0.000	0.248	0.015	0.564	0.000
C-SOMC-2	0.214	0.000	0.300	0.000	0.013	0.175	0.145	0.001	−0.042	0.638	−0.017	0.420
C-SOMC-4	0.348	0.000	0.302	0.000	−0.014	0.790	−0.015	0.797	0.225	0.020	0.222	0.021
C-SOMC-5	0.223	0.000	0.428	0.000	0.040	0.059	0.288	0.000	0.413	0.001	0.604	0.000
C-SOMC-6	0.049	0.023	0.134	0.000	0.017	0.152	0.176	0.000	E	E	E	E
C-SOMC-3/7	0.321	0.000	0.652	0.000	0.061	0.026	0.610	0.000	0.295	0.008	0.529	0.000
C-SOMC-R6	0.606	0.000	0.955	0.000	0.128	0.002	0.856	0.000	0.518	0.000	1.000	0.000

*P* < 0.05 indicates significant correlations. E:Because there is at least one constant, it cannot be statistics. C-SOMC, Chinese version of the Short Orientation-Memory-Concentration Test; C-SOMC-1, item 1 of the C-SOMC test; C-SOMC-2, item 2 of the C-SOMC test; C-SOMC-4, item 4 of the C-SOMC test; C-SOMC-5, item 5 of the C-SOMC test; C-SOMC-6, item 6 of the C-SOMC test; C-SOMC-3/7, item 3/7 of the C-SOMC test; C-SOMC-R6, five items (i.e., item 1, 2, 4, 5, and 3/7) of the C-SOMC test except item 6 constituted a shorter C-SOMC test; C-MMSE, Chinese version of the Mini Mental State Examination.

### True positive and true negative

The details of the true positives and true negatives using the three cut-offs (at 18/19, 17/18, and 16/17) for the total scores on the C-SOMC test and the two cut-offs (at 23/24 and 24/25) for the total C-MMSE scores are shown in Table 5. The results indicate that a cut-off at 17/18 for the total C-SOMC test against a cut-off at 23/24 for the C-MMSE score gave optimal performance, with 84.9% (73/86) of the participants’ score on the C-SOMC test classified in agreement with their C-MMSE score.

**TABLE 5:** Performance of the participants’ (*n* = 86) in the C-SOMC test against their performance in the C-MMSE.

	True positives (%)		True negative (%)		Agreement (%)		Sensitivity (%)		Specificity (%)	
**C-SOMC** **Cut-off**	**C-MMSE** **Cut-off**		**C-MMSE** **Cut-off**		**C-MMSE** **Cut-off**		**C-MMSE** **Cut-off**		**C-MMSE** **Cut-off**	
	24/25	23/24	24/25	23/24	24/25	23/24	24/25	23/24	24/25	23/24
18/19	68	75	85.2	83.3	80.2	81.4	68	75	85.2	83.3
17/18	64	75	88.5	87.7	83.7	84.9	64	75	88.5	87.9
16/17	52	60	90.2	89.4	79.1	82.6	52	60	90.2	89.4

C-SOMC, Chinese version of the Short Orientation Memory Concentration Test; C-MMSE, Chinese version of the Mini Mental State Examination.

### Sensitivity and specificity

The results demonstrated that a cut-off at 17/18 for the total score on the C-SOMC test against a cut-off at 23/24 for the C-MMSE score gave optimal performance, with 75% sensitivity and 87.9% specificity. Table 5 provides details of the performance of the C-SOMC test against the C-MMSE.

**FIGURE 2:**
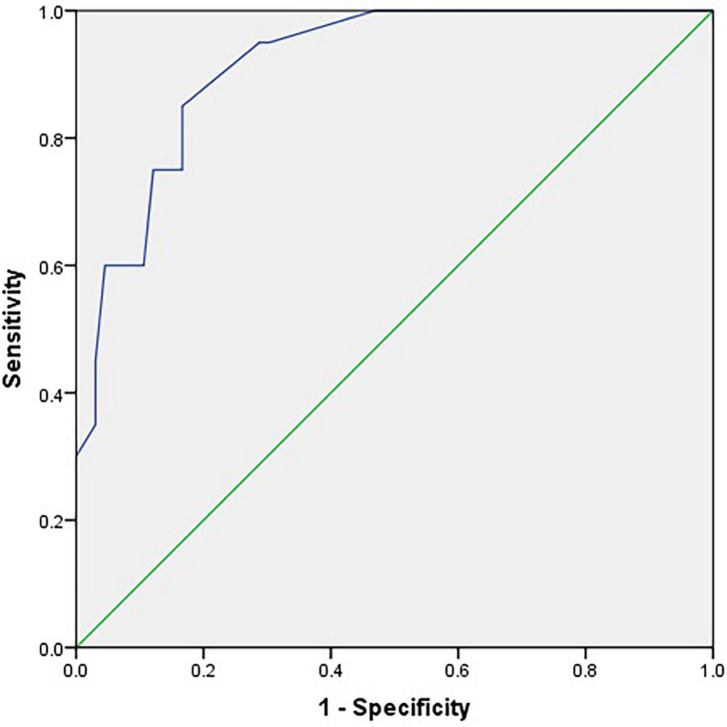
Receiver operating characteristic curve for the C-SOMC test using a cut-off at 23/24 on the C-MMSE in the stroke participants (*n* = 86).

**FIGURE 3:**
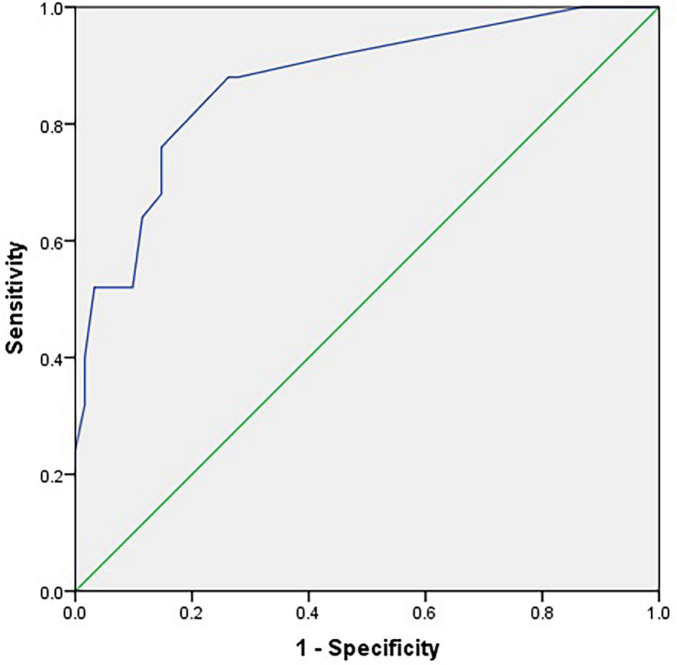
Receiver operating characteristic curve for the C-SOMC test using a cut-off at 24/25 on the C-MMSE in the stroke participants (*n* = 86).

The ROC curves for the C-SOMC test using cut-offs at 23/24 and 24/25 for the C-MMSE score are shown in Figures 2, 3, respectively. The AUCs were 0.92 and 0.87, respectively (*P <* 0.001, 95% confidence interval (CI) = 0.85 to 0.96; *P <* 0.001, 95% CI = 0.79 to 0.96).

## Discussion

This study was the first to translate the SOMC test into Chinese and adapt it to Chinese culture, forming the C-SOMC test. It was also the first study to investigate the concurrent validity, sensitivity, and specificity of the C-SOMC test in a Chinese population with a first cerebral infarction. The results showed that the participants’ performance on the C-SOMC test was well correlated with their performance on the C-MMSE. Additionally, a ROC analysis revealed that the C-SOMC test had good sensitivity and specificity, with a cut-off at 17/18 for the total score on the C-SOMC test against a cut-off at 23/24 for the score of C-MMSE giving optimal performance.

The validation analysis indicated that there was a significant correlation between the participants’ total scores in the C-SOMC test and their scores in the C-MMSE. Their scores for each of the six items in the C-SOMC test were also significantly correlated with their scores in the C-MMSE, with item 1 yielding the highest ρ-value and item 6 exhibiting the lowest ρ-value. The above-described findings suggest that the C-SOMC test is a valid measure for screening cognitive impairment in stroke patients, with validity comparable to that of the C-MMSE. Previous studies involving different populations have come to similar conclusions. For example, Davous et al. (1987) found a high correlation between the SOMC test and the MMSE in neurological, psychiatric, and dementia patients, demonstrating that the SOMC test and the MMSE are equivalently effective for identifying vascular or degenerative dementia. Similarly, Goring et al. (2004) showed that the SOMC test and the MMSE were highly correlated in assessment of patients in acute medical ward. Therefore, our study supports the validity of the C-SOMC test as a tool for screening cognitive impairment in stroke patients.

The total scores on the C-SOMC test and scores for each item in this test were good predictors of the C-MMSE score, except for item 6, which had a lower adjusted R^2^ than the other items. Furthermore, the scores for each of six items of the C-SOMC test were good predictors for the total score on this test, with the score for item 6 being the lowest. This might have been due to item 6 being the most difficult item for the participants to answer, leading to the variability in their responses to this item. That was, although all of the participants were very familiar with the TCZS, some of the participants only knew some of the order of the TCZS and could not state all of them quickly in the correct order.

As mentioned, item 6 of the C-SOMC test was deleted, and the remaining five items (items 1, 2, 4,5, and 3/7) constituted the C-SOMC-R6 test. The total score on the C-SOMC-R6 test was significantly correlated with the total score on the C-SOMC test (ρ = 0.933) and was a strong predictor of the total score on the C-SOMC test (adjusted *R*^2^ = 0.955). The total score on the C-SOMC-R6 test was also significantly correlated with the C-MMSE score (ρ = 0.624) and was a good predictor (adjusted *R*^2^ = 0.606) of the C-MMSE score. This suggested that the C-SOMC-R6 test may be a useful alternative to the C-SOMC test for screening cognitive function in stroke patients. However, compared with the corresponding values for correlation between the C-SOMC test and the C-MMSE, the ρ-value and adjusted *R*^2^ of the correlation between the C-SOMC-R6 test and the C-MMSE were slightly smaller. Therefore, further research is needed to confirm the validity of the C-SOMC-R6 test as a measure for screening cognitive impairment in stroke patients. For now, the C-SOMC test remains the optimal validated measure for this purpose.

We also found that the C-SOMC test had AUCs of 0.92 and 0.87 when using cut-offs at 23/24 and 24/25 on the C-MMSE, respectively. As an AUC of 0.80 or above is considered to indicate good accuracy (Goring et al., 2004), these results suggested that the C-SOMC test was highly accurate at distinguishing between cognitive impairment and normal cognition in the participants. A cut-off at 17/18 on the C-SOMC test against a cut-off at 23/24 on the C-MMSE yielded optimal performance: with 84.9% (73/86) of the participants were classified in agreement with the C-MMSE, with 75% sensitivity and 87.9% specificity. These findings are consistent with those of previous studies (Davous et al., 1987; Goring et al., 2004) and the recommendations from the original validation study (Katzman et al., 1983). In summary, our findings suggest that the C-SOMC test is a useful and sensitive tool for identifying cognitive impairment in patients with stroke.

This study had several limitations. First, the sample size was moderate, which may limit the generalizability of our findings. Second, 66 (76.74%) participants had normal cognition, whereas only 14 (16.28%) participants had a C-MMSE score of 18–23 and only six (6.98%) participants had a C-MMSE score of 0–17, indicating mild and severe cognitive impairment, respectively. This restricted our ability to analyze the C-SOMC test scores across different levels of cognitive impairment. Therefore, future studies should use a larger sample size and a broader range of cognitive impairment levels to further examine the performance of the C-SOMC test. Third, we did not compare the C-SOMC test scores of the participants with those of healthy individuals. Finally, we only assessed the concurrent validity of the C-SOMC test and did not evaluate other psychometric properties such as intra- and inter-rater reliability, responsiveness, and predictive validity. Hence, further research is required to comprehensively evaluate the validity and reliability of the C-SOMC test in stroke patients with varying degrees of cognitive impairment.

## Conclusion

The C-SOMC test appears to be a valid tool for assessing cognitive impairment in stroke patients. Our findings also demonstrate that the C-SOMC test is highly sensitive and specific, with performance levels similar to those of the C-MMSE. This suggests that the C-SOMC test can be effectively used in both clinical and research settings to evaluate Chinese stroke patients. However, further research should investigate the comprehensive psychometric properties of the C-SOMC test.

## Data availability statement

The original contributions presented in this study are included in the article/Supplementary material, further inquiries can be directed to the corresponding authors.

## Ethics statement

The studies involving human participants were reviewed and approved by the Medical Ethical Committee of the First Affiliated Hospital of Sun Yat-sen University [Ethics Number: (2014)88]. The patients/participants provided their written informed consent to participate in this study.

## Author contributions

D-FH, Y-RM, and J-LZ designed the experiment. J-LZ performed the experiment. J-LZ and P-MC analyzed the data and interpreted the results. J-LZ, P-MC, and SMN edited and wrote the manuscript. All authors reviewed and approved the final manuscript.
