# Supplementary material for: Translation and concurrent validity, sensitivity and specificity of Chinese version of Short Orientation Memory Concentration Test in people with a first cerebral infarction
Source: Front Hum Neurosci. 2023 Jun 1;17:977078. doi: 10.3389/fnhum.2023.977078 (PMC10268244; doi:10.3389/fnhum.2023.977078)
Supplement: Supplementary file 1 [file Table_1.docx]

Appendix 1

**中文版短期定向记忆注意力测试**

患者姓名： 住院号：

| 指令 日期 | | | | | |  |  |  |
| --- | --- | --- | --- | --- | --- | --- | --- | --- |
| 1. **今年是哪一年？** | | | | | 0 或4 分 |  |  |  |
|  |  |  |  |  | 患者答案 |  |  |  |
| 1. **现在是几月份？** | | | | | 0 或3分 |  |  |  |
|  |  |  |  |  | 患者答案 |  |  |  |
| 1. **重复下面的地址和称呼（选择一个）** | | | | | |  |  |  |
| a. 李伟 | b. 王军 | c. 张华 | | d. 刘波 | |  |  |  |
| 天河路42号 | 虹桥路34号 | 和平路26号 | | 长安街18号 | |  |  |  |
| 广州 | 上海 | 天津 | | 北京 | |  |  |  |
| **试着记住，在测试结束时会再问你。** | | | | | |  |  |  |
| 1. **现在大概是几点？**   （*误差在一个小时内。*） | | | | | 0 或3分 |  |  |  |
|  |  |  |  |  | 患者答案 |  |  |  |
| 1. **请从20倒数到1。**   （*每个错误扣2分。*） | | | | |  |  |  |  |
|  |  |  |  |  | 0 2 4分 |  |  |  |
| 20 19 18 17 16 15 14 13 12 11 10 9 8 7 6 5 4 3 2 1 | | | | | |  |  |  |
| 1. **请将十二生肖按照倒序说出来。**   （*每个错误扣2分。*） | | | | | 0 2 4分 |  |  |  |
| 猪 狗 鸡 猴 羊 马 蛇 龙 兔 虎 牛 鼠 | | | | |  |  |  |  |
| 1. **请重复刚才要你记住的人名和地址。**   （*每个错误扣2分。*） | | | 0 2 4 6 8 10分 | | |  |  |  |
|  |  |  | 所给地址（a, b, c, d） | | |  |  |  |
|  | | | **总分** | | | /28 | /28 | /28 |
