# Supplementary material for: Translation and concurrent validity, sensitivity and specificity of Chinese version of Short Orientation Memory Concentration Test in people with a first cerebral infarction
Source: Front Hum Neurosci. 2023 Jun 1;17:977078. doi: 10.3389/fnhum.2023.977078 (PMC10268244; doi:10.3389/fnhum.2023.977078)
Supplement: Supplementary file 2 [file Table_2.docx]

Appendix 2

**The Chinese version of the Short Orientation Memory and Concentration Test**

Patient's name： Hospital number：

| Instruction Date | | | | | |  |  |  |
| --- | --- | --- | --- | --- | --- | --- | --- | --- |
| 1. **What year is it now?** | | | | | Score 0 or 4 |  |  |  |
|  |  |  |  |  | Patient's answer |  |  |  |
| 1. **What month is it now?** | | | | | Score 0 or 3 |  |  |  |
|  |  |  |  |  | Patient's answer |  |  |  |
| 1. **Repeat this address (choose one):** | | | | | |  |  |  |
| a. Wei Li | b. Jun Wang | c. Hua Zhang | | d. Bo Liu | |  |  |  |
| 42/Tian He Road | 34/Hong Qiao Road | 26/ He Ping Road | | 18/Chang An Street | |  |  |  |
| Guangzhou | Shanghai | Tianjin | | Beijing | |  |  |  |
| **Try to remember this. I'll ask you to recall it at the end of the test.** | | | | | |  |  |  |
| 1. About what time is it?   （*within an hour.*） | | | | | Score 0 or 3 |  |  |  |
|  |  |  |  |  | Patient's answer |  |  |  |
| 1. Count backwards 20 down to 1**.**   （*Two points off for each error.*） | | | | |  |  |  |  |
|  |  |  |  |  | Score 0, 2, 4 |  |  |  |
| 20 19 18 17 16 15 14 13 12 11 10 9 8 7 6 5 4 3 2 1 | | | | | |  |  |  |
| 1. Say the Twelve Chinese Zodiac Sign in reverse order.   （*Two points off for each error.*） | | | | | Score 0, 2, 4 |  |  |  |
| Pig Dog Chicken Monkey Sheep Horse  Snake Dragon Rabbit Tiger Cow Rat | | | | |  |  |  |  |
| 1. **Repeat the address given.**   （*Two points off for each error.*） | | | Score 0, 2, 4, 6, 8, 10 | | |  |  |  |
|  |  |  | Address given（a, b, c, d） | | |  |  |  |
|  | | | **Total Score** | | | /28 | /28 | /28 |
